# Modified miR-15a has therapeutic potential for improving treatment of advanced stage colorectal cancer through inhibition of BCL2, BMI1, YAP1 and DCLK1

**DOI:** 10.18632/oncotarget.23414

**Published:** 2017-12-19

**Authors:** Andrew Fesler, Hua Liu, Jingfang Ju

**Affiliations:** ^1^ Department of Pathology, School of Medicine, Stony Brook University, Stony Brook, NY 11794, USA

**Keywords:** 5-fluorouracil, miR-15a, colorectal cancer, chemoresistance, modified miRNA

## Abstract

Despite advances in colon cancer treatments, resistance and recurrence remain a significant challenge in treating patients. Novel therapeutic strategies are in urgent need to overcome resistance and improve patient outcomes. MicroRNA based therapeutics have potential to help combat resistance. In this study, we have shown that low miR-15a expression correlates with poor patient prognosis. We have demonstrated the therapeutic potential of miR-15a in colon cancer. miR-15a inhibits several important genes (*BCL2, BMI1, YAP1* and *DCLK1*), decreasing cancer progression and resistance. Additionally, by replacing uracil in miR-15a with 5-fluorouracil, we created a novel miR-15a mimic with enhanced therapeutic potential. This mimic maintains target specificity and is more potent than unmodified miR-15a *in vitro* and inhibits colon tumor metastasis *in vivo*. This mimic has great potential for therapeutic development for treating colon cancer patients. This novel modification has potential to advance the development of other microRNA based therapeutics beyond miR-15a.

## INTRODUCTION

Despite improvements in patient care, colon cancer remains a clinical challenge. It is the 3^rd^ most common cancer diagnosed in the United States, and only 14% of patients with metastatic disease survive 5 years [1, 2]. Traditional chemotherapeutics such as 5-fluorouracil (5-FU) improve patient survival primarily through targeting rapidly proliferating cancer cells. 5-FU has been used for treatment of colon cancer for several decades [3]. However, resistance to 5-FU and recurrence following treatment remains a challenge. 40% of patients treated with 5-FU based therapy following surgery experience recurrence and die within 8 years, and only around 50% of patients are responsive to 5-FU combination therapies [4, 5]. Thus there is a need to identify novel therapeutic strategies to enhance treatment efficacy. The challenges of resistance and recurrence involve multiple mechanisms and are associated with the presence of a subpopulation of colon cancer stem cells (CSC) [6–9]. These colon CSCs are resistant to traditional chemotherapeutics due to their slow proliferation, inhibition of apoptosis, and increased Wnt signaling [10–12]. To overcome resistance and recurrence, we need to develop novel therapeutic agents that are effective against colon CSCs. One approach to preventing chemoresistance is to hit multiple targets that regulate different cellular pathways involved in resistance. This can be achieved through the utilization of microRNA (miRNA) based therapeutics.

miRNAs are short non-coding RNAs that regulate the expression of target genes through interaction with the 3’UTR of target mRNA [13]. This leads to inhibition of target protein expression by degradation of the mRNA or by translational inhibition [14, 15]. One miRNA can inhibit a number of different mRNAs. This makes miRNAs an intriguing therapeutic option, as they provide a means to inhibit several genes at once which may be helpful in combating resistance. In combination with traditional chemotherapeutics, the effects of the miRNA may help to eliminate cells that would otherwise be resistant to chemotherapy.

One of the first miRNAs identified to have a role in cancer, is miR-15a. Deletion of the locus containing miR-15a is common in chronic lymphocytic leukemia (CLL) patients [16]. In CLL and other cancers, anti-apoptotic *BCL2* has been identified as a target of miR-15a [17]. miR-15a also targets *BMI1*, a stem cell marker and promoter of invasion and migration, in gastric cancer as well as pancreatic cancer [18–21]. With these two genes having important, anti-apoptotic and EMT promoting roles in colon cancer, suppression of these genes by miR-15a in colon cancer may be helpful for therapeutic intervention. This makes miR-15a a promising miRNA for investigation of its therapeutic potential.

In this study, we have used the The Cancer Genome Atlas (TCGA) database to analyze the expression of miR-15a in colon cancer patient samples and found a positive correlation between miR-15a expression and patient prognosis. In addition, we have shown that miR-15a inhibits the previously described targets *BCL2* and *BMI1* in colon cancer as well as novel targets, *YAP1*, a transcriptional coactivator and oncogene and *DCLK1*, a colon CSC marker [22–27]. We have also found that ectopic expression of miR-15a in colon cancer cells inhibits proliferation, and increases the effectiveness of 5-FU. In addition, in stem like colon cancer cells, miR-15a reduces sphere formation *in vitro* and decreases tumor formation and growth *in vivo*. In investigating the therapeutic potential of miR-15a, we have created a novel modified miR-15a mimic that combines the functions of miR-15a with that of 5-FU. This modified mimic demonstrates enhanced ability to disrupt colon cancer cell growth and metastasis, as well as therapeutic potential *in vivo*. This mimic is a strong candidate for therapeutic development.

## RESULTS

### The expression of miR-15a is significantly associated with patient survival

**Figure 1:**
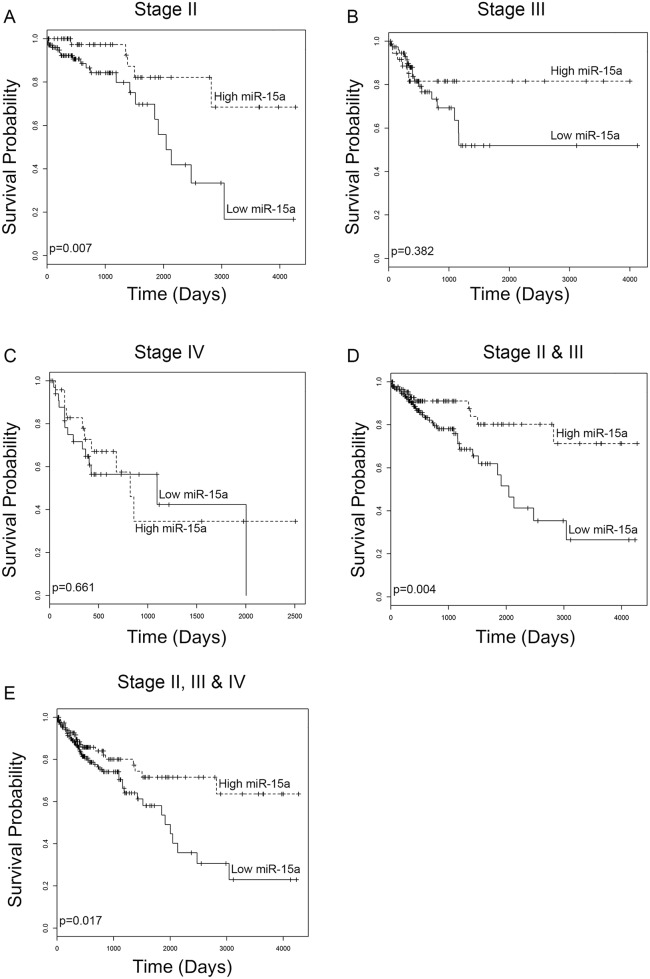
miR-15a expression analysis from TCGA database Low expression of miR-15a correlates with worse patient survival for patients with stage II colon cancer **(A)** (*p*=0.007). Low expression associates with worse survival for patients with stage III colon cancer, however the difference is not statistically significant **(B)** (*p*=0.382). There is no correlation between miR-15a expression and survival of patients with stage IV cancer **(C)** (*p*=0.661). Low expression of miR-15a correlates with decreased survival for combined analysis of stage II & III patients **(D)** (*p*=0.004) as well as stage II, III & IV patients **(E)** (*p*=0.017).

In order to assess the relationship between miR-15a expression and survival in colon cancer patients, we utilized TCGA RNA sequencing dataset for colorectal cancer. Survival analysis was performed for stage II, III, and IV patients as well as combinations of patients from all stages. This analysis indicates that for stage II patients, low expression of miR-15a is significantly (*p*=0.007) correlated with shorter survival (Figure 1A). In stage III patients, low expression of miR-15a is associated with shorter survival however the difference is not statistically significant (*p*=0.382) (Figure 1B). In stage IV patients, there is no correlation between survival and miR-15a expression (*p*=0.661) (Figure 1C). When patients from different stages are grouped for analysis, a significant correlation between low miR-15a expression and worse survival is found for the group containing stage II & III (*p*=0.004) (Figure 1D) and all patients (II, III & IV) (*p*=0.017) (Figure 1E).

### Identification and validation of BCL2, BMI1, YAP1 and DCLK1 as key targets of miR-15a

**Figure 2:**
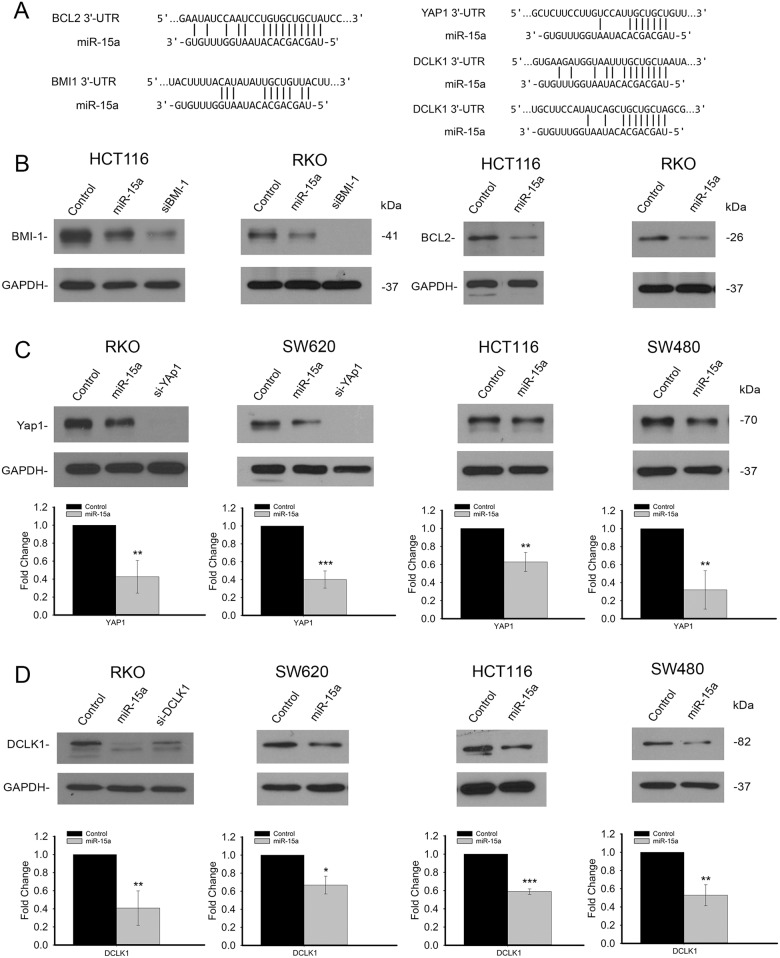
miR-15a inhibits several important targets in colon cancer miR-15a binding sites are found in the 3’UTR's of 4 genes important in colon cancer, *BCL2, BMI1, YAP1 and DCLK1* **(A)**. miR-15a expression in HCT116 and RKO cell lines reduced protein expression of BMI1 and BCL2 **(B)**. Western blot analysis of YAP1 expression following transfection with miR-15a in 4 different colon cancer cell lines shows decreased YAP1 expression. Representative Western blots demonstrating a decrease in YAP1 protein expression and quantification of protein expression is shown **(C)**. Western blot analysis of DCLK1 expression following transfection with miR-15a in 4 different colon cancer cell lines shows decreased DCLK1 expression. Representative Western blots demonstrating a decrease in DCLK1 protein expression and quantification of protein expression is shown **(D)**. (^*^ *p ≤* 0.05, ^**^ *p ≤* 0.01, ^***^ *p ≤* 0.001).

The significant clinical association of miR-15a with colorectal cancer patient survival established the importance of miR-15a in colorectal cancer. To further understand the molecular and cellular mechanism of miR-15a, we investigated the function of miR-15a in colon cancer cells. Several important colon cancer related genes, including well established targets of miR-15a, *BCL2* and *BMI1* as well as novel targets, *YAP1* and *DCLK1* contain binding sites for miR-15a in their 3’UTRs (Figure 2A). To assess the effect of miR-15a on the expression of these genes, miR-15a was overexpressed by transient transfection in colon cancer cell lines. Western blot analysis showed that protein expression of both BCL2 and BMI1 is reduced by miR-15a in HCT116 and RKO cells (Figure 2B). In addition to determining that these well-established miR-15a targets from other cancers are targets in colon cancer, we identified novel targets using target prediction algorithms (TargetScan). Western blotting showed a reduction of YAP1 protein expression following miR-15a over expression in all four cell lines tested. Quantification of the reduction of YAP1 protein expression indicated that the reduction was significant and ranged from 40% to 70% for the different cell lines studied (Figure 2C). DCLK1 protein expression also decreased following over expression of miR-15a. Quantification of the protein expression of DCLK1 indicated that the decrease was significant and ranged from 35% to 60% in the different cell lines studied (Figure 2D).

**Figure 3:**
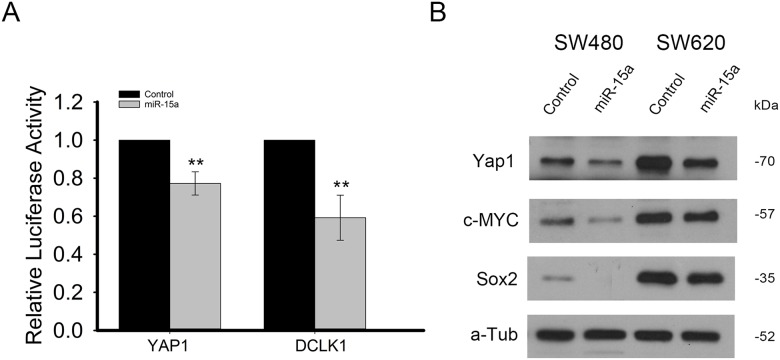
miR-15a directly targets YAP1 and DCLK1 and inhibits expression of genes downstream of YAP1 Luciferase reporter assay in HCT116 cells, demonstrates that miR-15a expression can decrease luciferase expression by reporter vectors including binding sites for miR-15a from the YAP1 and DCLK1 3’UTRs, indicating direct targeting of YAP1 and DCLK1 by miR-15a **(A)**. miR-15a expression reduced protein expression of downstream targets of YAP, c-MYC as well as SOX2 in SW480 and SW620 cell lines **(B)**. (^**^ *p ≤* 0.01).

In order to confirm that miR-15a directly targets *YAP1* and *DCLK1*, a portion of both of their 3’UTRs which included predicted miR-15a binding sites were cloned into luciferase reporter vectors. Following transfection of both the reporter vector and either miR-15a or control miRNA in HCT116 cells, results showed that luciferase activity was significantly reduced for both, indicating direct targeting by miR-15a for both *YAP1* and *DCLK1* (Figure 3A). We further investigated the expression of some genes shown to be downstream of *YAP1* and found that miR-15a overexpression decreased the protein expression of both SOX2 and c-MYC (Figure 3B).

### miR-15a decreases colon cancer cell proliferation and inhibits cell cycle progression

**Figure 4:**
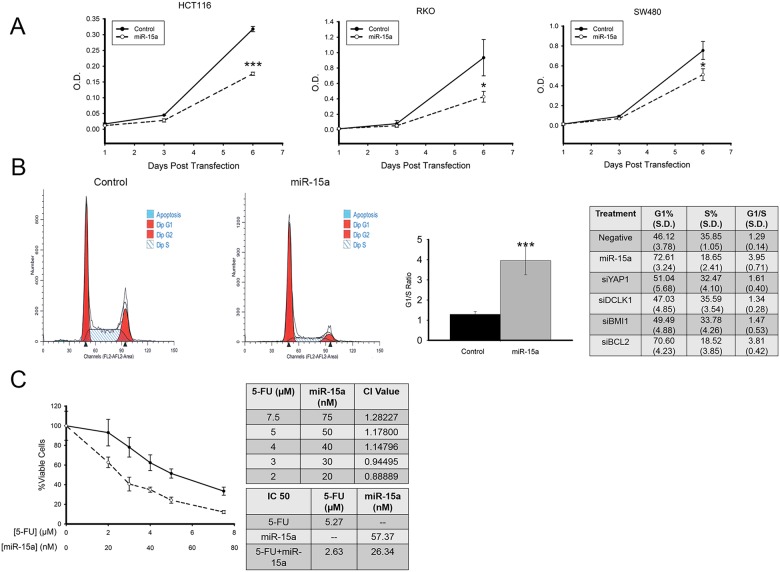
miR-15a inhibits colon cancer proliferation, and increases sensitivity to 5-Fu miR-15a reduces colon cancer cell proliferation measured by WST-1 assay in 3 colon cancer cell lines, HCT116, RKO, SW480 **(A)**. miR-15a expression induces cell cycle arrest in HCT116 colon cancer cells. Representative flow cytometry patterns for cells transfected with control or miR-15a and the effects of miR-15a and siRNA against its target genes on G1%, S-Phase % and G1/S ratio are shown **(B)**. miR-15a expression increases sensitivity to 5-FU in HCT116 colon cancer cells. CI values calculated for miR-15a and 5-FU indicate at low concentrations they have a slight synergistic effect. In combination, the IC^50^ of 5-FU was reduced from 5.27 to 2.63 μM, and the IC^50^ of miR-15a was reduced from 57.37 to 26.34 nM **(C)**. (^*^ *p ≤* 0.05, ^***^ *p ≤* 0.001).

With these important targets of miR-15a, (*BCL2*, *BMI1*, *YAP1* and *DCLK1*) in colon cancer having potential to promote colon cancer growth, we investigated the effects of miR-15a on cell proliferation. Using colon cancer cells transfected with miR-15a or control miRNA, WST-1 dye was used to assess cell number, 1, 3 and 6 days post transfection. In colon cancer cell lines HCT116, RKO and SW480, there was a reduction in the number of cells 6 days after transfection (Figure 4A). miR-15a had minimal effect on proliferation of normal colon cancer cells (data not shown). We also performed cell cycle analysis to determine the effects that miR-15a has on cell cycle progression. Representative flow cytometry results for control transfected cells, and for miR-15a transfected cells are shown in Figure 4B. Transfection of miR-15a results in a significant increase in the G1/S ratio for colon cancer cells. We also tested siRNA against the different target genes to determine the role that the knockdown of each of these genes has on cell cycle progression. The largest increase in G1/S ratio was seen for si-*BCL2* (Figure 4B).

### miR-15a enhances the therapeutic effect of 5-FU on colon cancer cells

5-FU based therapy has been the standard treatment for metastatic colon cancer for several decades. Thus we were curious to investigate the effect that a combination of miR-15a expression along with 5-FU treatment would have on colon cancer cells. To test this, HCT116 cells were treated with 5-FU alone at different concentrations ranging from 2 μM to 7.5 μM, transfected with various concentrations of miR-15a (20 nM to 75 nM), or a combination of the two in a fixed 1:100 ratio. Three days after treatment, cell viability was determined using WST-1 dye. Results indicated that the combination of miR-15a expression with 5-FU treatment lead to a greater decrease in cell viability compared to 5-FU alone (Figure 4C). Analysis with CombuSyn indicated that at lower concentrations, there was a weak synergistic effect of miR-15a in combination with 5-FU (CI<1). In addition, the IC^50^ of miR-15a alone was calculated at 57.37 nM but was reduced to 26.34 nM in combination with 5-FU. The IC^50^ for 5-FU alone was calculated at 5.27 μM, and in combination with miR-15a, was reduced to 2.63 μM (Figure 4C). Thus, expression of miR-15a enhances the effectiveness of 5-FU therapy.

### Overcoming resistance of colon cancer stem cells by miR-15a

**Figure 5:**
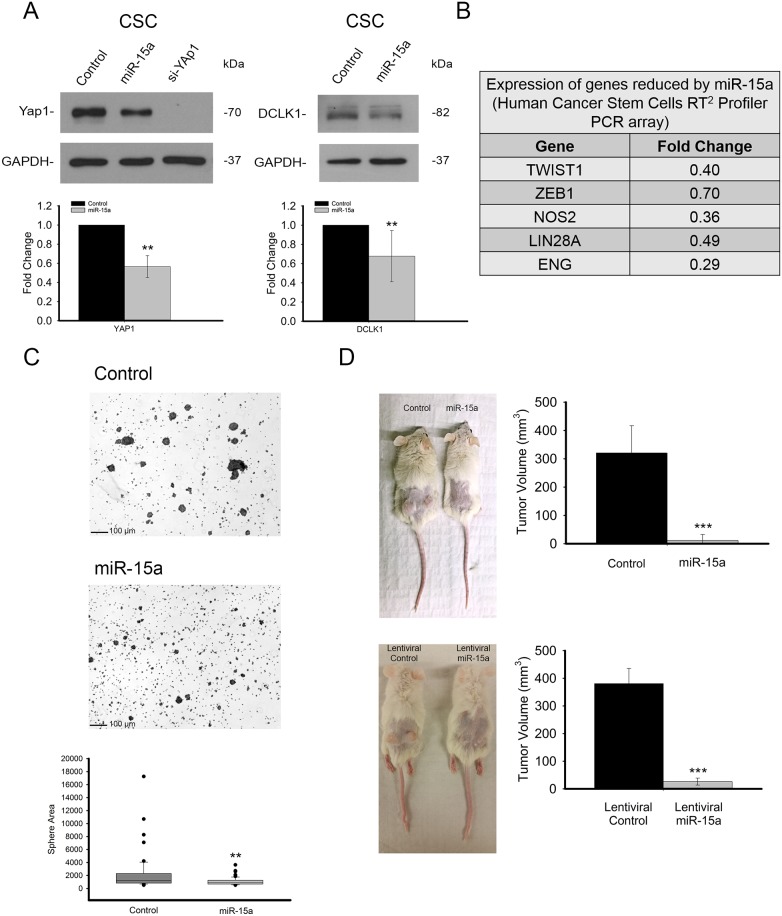
miR-15a is effective in spheroid growing stem like HCT116 colon cancer cells and inhibits tumor formation *in vivo* In spheroid growing cells, expression of miR-15a also reduces expression of YAP1 and DCLK1 **(A)**. A PCR array looking at cancer stem cell related genes indicates that miR-15a expression leads to changes in a group of cancer stem cell related genes including *TWIST1* and *ZEB1* **(B)**. miR-15a expression inhibits the ability of these cells to form spheres and sphere size was quantified **(C)**. miR-15a expression by transient transfection prior to tumor implantation (top), or stably via lentiviral vector (bottom) drastically reduces tumor formation and growth in NOD/SCID mice **(D)**. (^**^ *p ≤* 0.01, ^***^ *p ≤* 0.001).

Colon CSCs represent a major clinical challenge as these cells are resistant to traditional chemotherapeutic treatment and may be responsible for recurrence. We were curious to determine the effect that miR-15a may have on spheroid growing HCT116 cells which have been shown to be stem like and resistant to 5-FU [28, 29]. We first checked that expression of the miR-15a targets, YAP1 and DCLK1 was reduced in these cells upon miR-15a over expression. Western blot revealed that the expression of both proteins was significantly reduced in spheroid cells as a result of miR-15a expression (Figure 5A). In order to get a broad picture of the effects of miR-15a expression in these stem like cells, we used a CSC marker RT-PCR array to see what CSC related genes were affected by miR-15a. Some genes such as *TWIST* and *ZEB1*, both important in EMT, which exhibited significant reduction in the cells transfected with miR-15a are shown in Figure 5B. A full list of expression fold changes for all of the genes from this array is included in Supplemental Table 1. Functionally, we also observed that miR-15a transfection inhibits the ability of these cells to form spheres. Representative photos and sphere size quantification are shown in Figure 5C.

### miR-15a inhibits colon cancer stem cell mouse xenografts

To further our understanding of miR-15a in spheroid growing, stem like HCT116 colon cancer cells, we established mouse xenografts with these cells that were either pre-transfected with miR-15a or control miRNA, as well as stably expressing miR-15a from lentiviral vector or vector control virus. Eight weeks after injection, tumors were measured and harvested. We found a drastic reduction in tumor size for tumors established from cells expressing miR-15a via transient transfection (>25x) (n=8) as well as via lentiviral vector (>10X) (n=4) (Figure 5D).

### Modified miR-15a mimic exhibits enhanced therapeutic potential *in vitro* and *in vivo*

**Figure 6:**
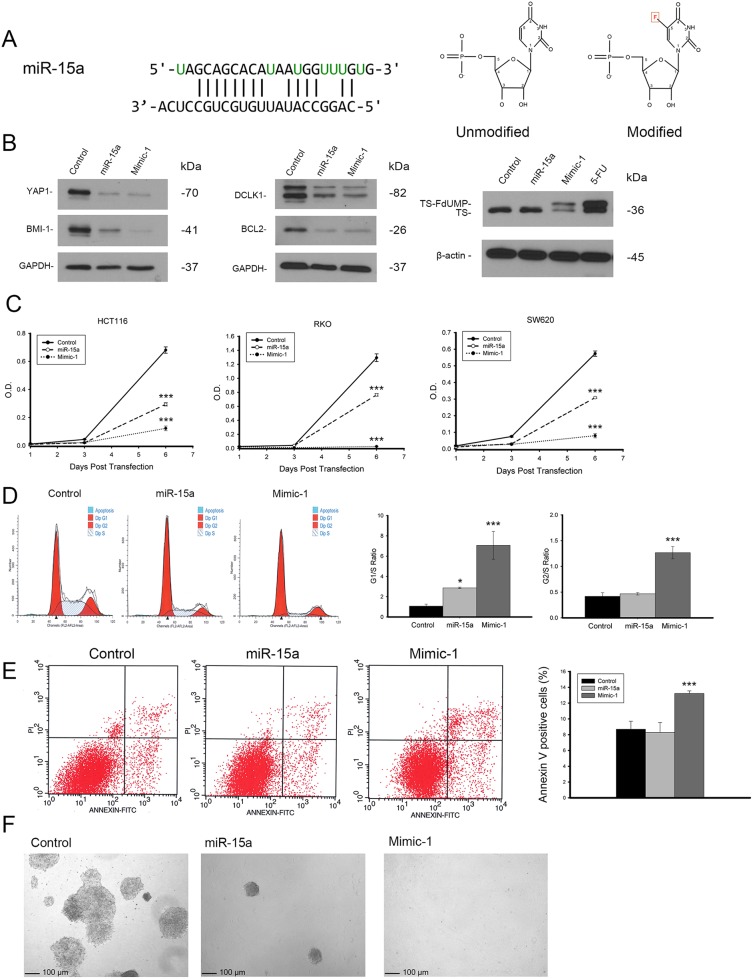
Modified miR-15a mimic is more potent than miR-15a and retains target specificity Modified miR-15a mimic, Mimic-1 was made in which all uracils in miR-15a were replaced with 5-FU. The structure of unmodified uracil vs 5-FU is shown **(A)**. Mimic-1 was shown to maintain the ability to regulate protein expression of miR-15a targets and also act as 5-FU shown by the presence of TS-FdUMP **(B)**. Mimic-1 showed enhanced ability to inhibit colon cancer cell (HCT116, RKO and SW620) proliferation compared to unmodified miR-15a **(C)**. Mimic-1 also induced enhanced cell cycle arrest compared to unmodified miR-15a as shown by increased G1/S ratio **(D)**. Mimic-1 induced a 1.5 fold increase in apoptotic cells compared to control treated HCT116 cells based on Annexin V staining **(E)**. In spheroid growing HCT116 cells, unmodified miR-15a inhibited colony formation in 3D Matrigel matrix, while Mimic-1 prevented colony formation completely **(F)**. (^**^ *p ≤* 0.01, ^***^ *p ≤* 0.001).

**Figure 7:**
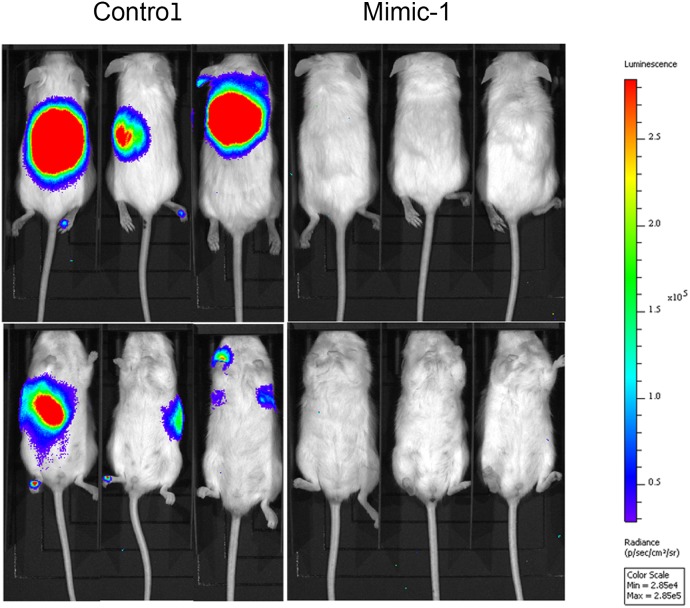
Mimic-1 is effective *in vivo* In metastatic mouse tumor models, established via tail vein injection of HCT116 colon cancer cells, Mimic-1 treatment via tail vein injection prevented metastatic tumor formation (n=5).

In order to enhance the therapeutic potential of miR-15a, we made a modified miR-15a mimic (Mimic-1) in which all of the uracil residues in the guide strand are replaced with 5-FU (Figure 6A). In testing the effectiveness of Mimic-1, we first determined that it retained target specificity. Western blot analysis following transfection of Mimic-1 in HCT116 derived spheroid growing cells demonstrates that Mimic-1 retains the ability to reduce expression of targets YAP1, BMI1, DCLK1, and BCL2 (Figure 6B). We also assessed the effects of 5-FU alone on the target genes and found no significant changes in YAP1, BMI1, DCLK1 and BCL2 expression (Supplemental Figure 1). The detection of the thymidylate synthase FdUMP (TS-FdUMP) complex by Western blot also confirmed that Mimic-1 released functional 5-FU into cells (Figure 6B). In addition, Mimic-1 had an enhanced ability to inhibit cell proliferation compared to miR-15a (Figure 6C). We hypothesized that this may be the effect of increased cell cycle arrest associated with Mimic-1 compared to miR-15a. Mimic-1 transfection results in a substantial increase in both the G1/S and G2/S ratio (Figure 6D). Mimic-1 also induced a 1.5 fold increase in apoptotic cells compared to control (Figure 6E). We confirmed that the effects of Mimic-1 were not due to 5-FU alone using a non-targeted *C. elegans* control miRNA, *cel-miR-67,* with 5-FU modifications, which reduced cell number 40% compared to 95% for Mimic-1 six days after transfection (Supplemental Figure 2). In HCT116 derived spheroid growing cells, while miR-15a inhibited the ability of these cells to form colonies in Matrigel matrix, Mimic-1 prevented these cells from forming colonies (Figure 6F). In a mouse metastasis model in which HCT116 colon cancer cells were injected via the tail vein, Mimic-1 inhibited metastatic tumor formation when delivered systemically through tail vein injection using *in vivo*-jetPEI (Figure 7). These mice exhibited no signs of toxicity from Mimic-1. Another group of mice was treated with 50 mg/kg 5-FU however these mice were not imaged as they died from 5-FU toxicity before the completion of the experiment.

### Vehicle free delivery of Mimic-1 to colon cancer cells

**Figure 8:**
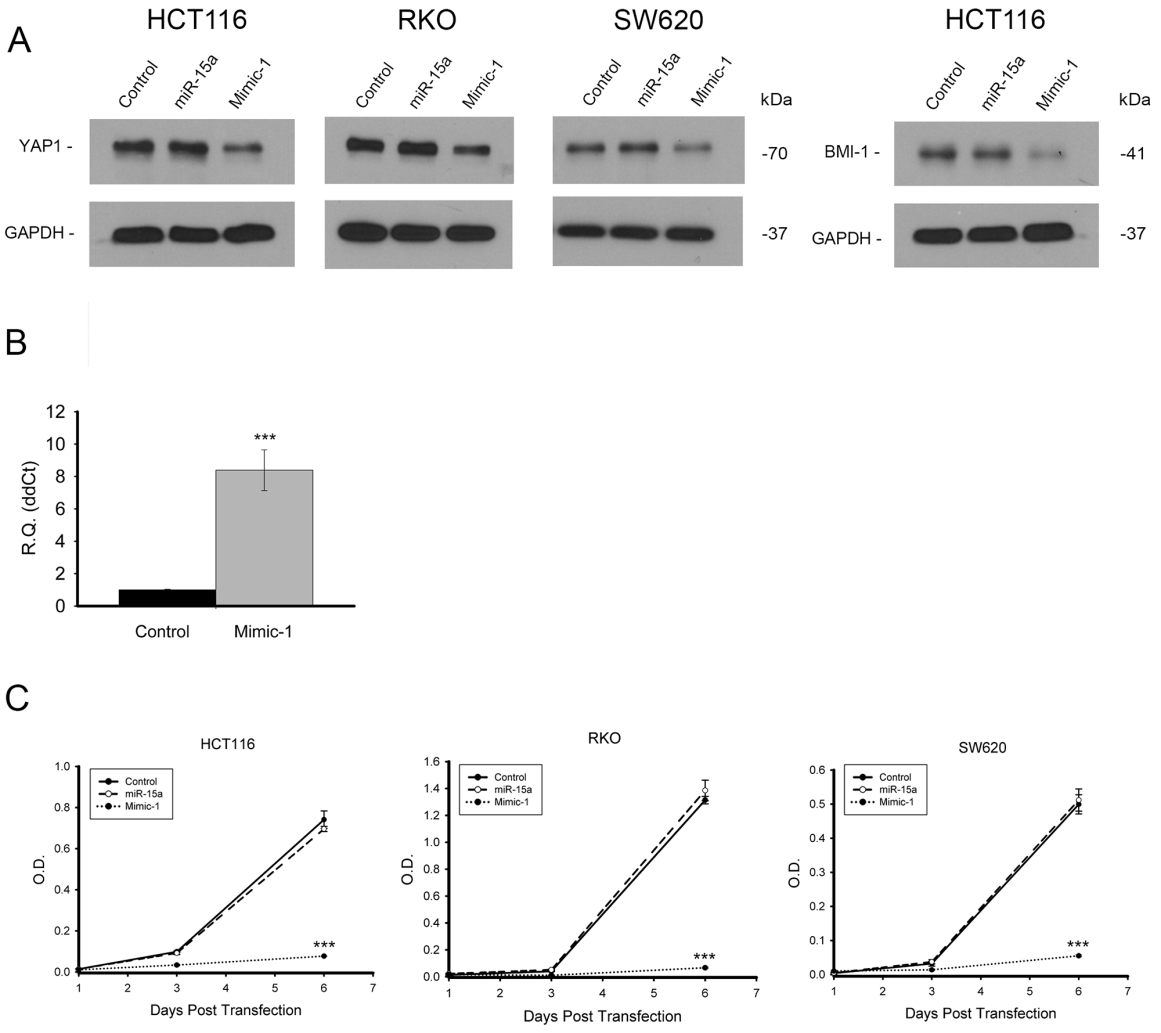
Vehicle free delivery of Mimic-1 *in vitro* Mimic-1, when delivered without transfection reagents into colon cancer cells reduced protein expression of targets YAP1 and BMI1 **(A)**. qRT-PCR confirmed that miR-15a was entering HCT116 cells without transfection reagents **(B)**. In the absence of transfection reagents, Mimic-1 maintains its ability to decrease colon cancer cell proliferation, (HCT116, RKO and SW620) while unmodified miR-15a has no effect on cell proliferation **(C)**. (^***^ *p ≤* 0.001).

Generally for transient transfection experiments, miRNA are delivered into cells using lipid based transfection reagents. However, Mimic-1 delivery into colon cancer cells is possible without any transfection reagent. When 100 pmol of control miRNA, miR-15a or Mimic-1 was added to colon cancer cells growing in 2 ml regular growth medium (50 nM), we found that protein expression of target genes YAP1, and BMI1 was reduced in cells three days after treatment with Mimic-1, but not in cells treated with miR-15a (Figure 8A). We confirmed the ability of Mimic-1 to enter cells without a trasnfection reagent using qRT-PCR (Figure 8B). In addition, when we investigated cell proliferation, we found that Mimic-1 without transfection reagents drastically reduced cell number 6 days after transfection, while unmodified miR-15a alone had no effect (Figure 8C).

## DISCUSSION

**Figure 9:**
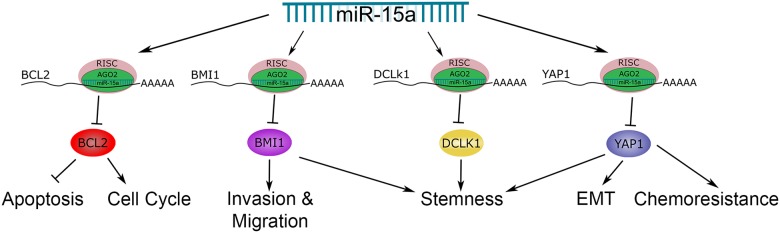
The role of miR-15a in colon cancer This schematic highlights the target genes of miR-15a, BCL2, BMI1, DCLK1 and YAP1 that we have investigated and the roles they play in colon cancer.

In this study, we have shown loss of miR-15a expression correlates with poor prognosis in advanced stage colon cancer patients, demonstrating the clinical relevance of miR-15a in colon cancer. We have also identified several important targets of miR-15a in colon cancer, *BCL2*, *BMI1*, *YAP1*, and *DCLK1*. *BCL2* is an inhibitor of apoptosis in colon cancer. Its expression has been shown to be important in intestinal cell transformation and development of 5-FU resistance [30–32]. *BMI1* is an oncogene with elevated expression in colon cancer patients that is important for self-renewal of colon CSCs and promotes invasion and migration of colon cancer cells [21, 33, 34]. *BMI1* expression is associated with stem like cells in the intestinal epithelium [35]. *BMI1* interacts with several signaling pathways including Notch and Wnt, which are important in colon cancer and chemoresistance [36]. *YAP1* is a transcriptional coactivator that is the downstream target of the Hippo signaling pathway [37]. In colon cancer patients, *YAP1* expression correlates with poor prognosis and chemoresistance [38]. *YAP1* plays an important role in regulating organ development and tissue size. In the mouse intestine, *YAP1* is expressed in intestinal stem cells, and *YAP1* activation promotes expansion of undifferentiated cell populations [39]. There is also interaction between *YAP1* and the Wnt signaling pathway. Furthermore, *YAP1* can regulate the expression of many different genes by interacting with several transcription factors including TEAD and SMAD [37]. In colon cancer, *YAP1* promotes invasion, migration, cell growth and EMT [23, 40]. *YAP1* regulates the expression of *SOX2* and *c-MYC*, which are overexpressed in colon cancer and promote metastasis and tumor growth [27, 41–43]. *DCLK1* is expressed in stem cells of normal colon tissue and has been identified as a marker of colon CSCs [44, 45]. Its expression is associated with metastasis and poor prognosis in colon cancer patients [46]. All of these genes have important functions in colon cancer and are potential therapeutic targets. Thus it is significant that miR-15a can suppress these four different targets, regulating a network of oncogenes (Figure 9).

In addition, we have found that expression of miR-15a in colon cancer cells reduces proliferation, induces cell cycle arrest and enhances the effectiveness of 5-FU. In mouse models miR-15a expression reduced tumor formation and growth suggesting therapeutic potential for miR-15a. It is likely through the suppression of the oncogenes we have identified in this study, as well as additional targets, that miR-15a contributes to increased survival in patients and can carry out the therapeutic effects we have demonstrated [47]. A schematic of the miR-15a target genes we have investigated and the cancer related pathways they regulate is shown in Figure 9. In regulating a group of genes, miR-15a based therapeutics have potential to avoid problems associated with resistance that are often seen with targeted therapies. By targeting a network of oncogenes it should be more difficult for the cancer to develop resistance.

Our initial goal was to determine if restoration of miR-15a expression could be a therapeutic strategy in colon cancer. With the promising results for miR-15a, we then sought to determine if we could enhance the therapeutic effects of miR-15a. With this aim, we generated a miR-15a mimic, Mimic-1, in which all of the uracil bases in the miR-15a guide strand are replaced with 5-FU. In designing Mimic-1 we hoped to not only increase the therapeutic potential of miR-15a but also preserve its miRNA function. Previous work has shown that 5-FU incorporation into messenger RNA does not disrupt mRNA translation or other characteristics including base pairing, suggesting that incorporation into miRNA should not disrupt its function [48]. Mimic-1 should inhibit miR-15a target genes and, once broken down release 5-FU into the cell. In this way the cell is hit by both the regulation of the miRNA and the effects of 5-FU. In using Mimic-1 in colon cancer cells, we found it had greatly enhanced effects on reducing proliferation compared to unmodified miR-15a. Importantly, we also found that target gene regulation was retained by Mimic-1. The ability of the 5-FU residues in Mimic-1 to act as 5-FU was confirmed by the presence of the TS-FdUMP complex [49]. We also confirmed the effects were not caused by 5-FU alone using a *C. elegans* control miRNA incorporating the 5-FU modification. Thus as we hoped, Mimic-1 combines the effects of miR-15a, target gene suppression, with those of 5-FU, TS inhibition and DNA damage, making it more potent at inducing cell cycle arrest and suppressing proliferation in cancer cells.

In mouse models of metastatic colon cancer, we found that systemic delivery of Mimic-1 inhibited metastatic tumor formation, confirming its therapeutic potential. Importantly, these mice showed no signs of toxicity from Mimic-1 such as weight, appetite or hair loss. This may be a result of miR-15a having little effect on normal cells, and the low dose of 5-FU associated with Mimic-1. This is in contrast to mice treated with 5-FU which died before the experiment was completed from the toxic effects of 5-FU. These findings strongly suggest a potential therapeutic application for Mimic-1.

The data presented here supports the viability of a novel modification in which 5-FU is incorporated into a miRNA to enhance its cytotoxicity for therapeutic function. This concept has been investigated in siRNA, with the incorporation of 5-fluoro-2’-deoxyuridine into siRNA against TS [50]. This study also demonstrated enhanced cytotoxicity of the modified siRNA. Interestingly, when this modification was made in the guide strand, the ability of the siRNA to silence the target gene was disrupted, and the modifications had to be incorporated into the passenger strand to maintain target regulation. We did not observe this for Mimic-1, where 5-FU residues were incorporated into the guide strand, and target regulation was maintained. This discrepancy between the effects in siRNA compared to miRNA may be a result of the dependence of siRNA on perfect base pairing and mRNA cleavage while miRNA typically have imperfect base pairing with the target and commonly disrupt translation in the absence of mRNA degradation. In the case of miR-15a, there are no uracil residues in the seed sequence, which is most important for target binding.

In addition to the enhanced therapeutic potential we discovered for our modified miR-15a mimic, we also determined that Mimic-1 can be transfected into colon cancer cells without a transfection reagent. In the presence or absence of transfection reagent, Mimic-1 has a similar effect on cell proliferation and target regulation. In contrast, without transfection reagents, unmodified miR-15a has no effect on cell proliferation or target expression. This is strong evidence that the 5-FU substitution enables Mimic-1 to cross the cell membrane and enter the cell, a novel and intriguing feature of this modification. There has been some previous evidence for the effectiveness of naked siRNA delivery *in vivo* [51]. Vehicle free delivery has also been demonstrated in locked nucleic acid (LNA) antisense studies (gymnosis) [52]. Other modifications have been investigated for vehicle free delivery of oligonucleotides, but these have focused on lipid based modifications [53]. The mechanism by which the incorporation of 5-FU enables the miRNA to enter the cell needs to be thoroughly investigated. Based on concerns about the stability and tissue distribution of naked Mimic-1 *in vivo*, as well as its ability to cross the blood brain barrier, we used *in vivo*-jetPEI, which has been shown to be effective for miRNA delivery *in vivo* to demonstrate the *in vivo* therapeutic potential in this study [54]. The *in vivo* potential of vehicle free delivery of Mimic-1 needs to be investigated.

Our work has demonstrated that miR-15a plays an important role in colon cancer. Restoration of miR-15a may have therapeutic potential for treatment of colon cancer patients, as it inhibits colon cancer cell proliferation, stemness and increases sensitivity to 5-FU. In the interest of developing the therapeutic application of miR-15a, we have shown that substitution of uracil with 5-FU in miR-15a drastically enhances its therapeutic potential. This modification combines the effects of miR-15a with 5-FU to increase the cytotoxicity of miR-15a. With additional investigation, Mimic-1 has great potential for development as a colon cancer therapeutic. This modification may be applicable to other miRNAs to enhance their therapeutic potential, and may be an important advancement for the development of effective miRNA therapeutics.

## MATERIALS AND METHODS

### TCGA survival analysis

In this study, we extracted the clinical, and miRNA expression data for TCGA colon adenocarcinoma from the UCSC cancer genome browser [55]. The miRNA expression was measured using HiSeq platform, and genome-wide characterizations of their expression patterns have been reported previously [56]. For the clinical data, the survival information for 431 subjects is available and the details can be found on TCGA database. There are 331 subjects that have both survival and miRNA expression data. We performed overall survival analyses for stage II, III & IV colon adenocarcinoma patients separately as well as in combination. We dichotomized the expression profile of miR-15a into two groups (high, low). The cut-off is 65%, that is, expression greater than the 65 percentile of expression of the patients was denoted as “high”; otherwise it was denoted as “low”. Subsequently, we performed log-rank tests between two groups and generated corresponding Kaplan–Meier curves.

### Cell lines and transfection

The human colon cancer cell lines HCT116, RKO, SW480, SW620 and the normal colon cell line CCD 841 CoN, were obtained from the American Type Culture Collection (ATCC) and maintained in McCoy's 5A medium (HCT116), DMEM (RKO, SW480, SW620) and MEM (CCD 841 CoN) (Thermo Fischer). Media was supplemented with 10% fetal bovine serum (Thermo Fischer). For Transfection, 1×10^5^ cells were plated in six-well plates and transfected with 100 nM of either miR-15a, non-specific control miRNA (Thermo Fischer) or modified miR-15a mimics (Dharmacon) after 24 hours, using Oligofectamine (Thermo Fischer) following the manufacturer's protocols. For reagent free transfection, cells were plated in 6 well plates at 1×10^5^ cells per well. Twenty-four hours later 100 pmol miRNA (Control, miR-15a, Mimic-1) were diluted in Optimem (Thermo Fischer) and added to the plate. Media was changed after 24 hours.

Stem like colon cancer cells were obtained as described previously [8, 29, 57]. For transfection, spheres were dissociated and single cell suspensions were plated in ultra low attachment 6-well plates at 2×10^5^ cells per well. Transfection was performed using Lipofectamine 2000 (Thermo Fischer) following the manufacturer's protocols.

### Western immunoblot analysis

Equal amounts of protein (15 μg) extracted from cells lysed in RIPA buffer with protease inhibitor (Sigma) were separated on 10%-12% sodium dodecyl sulfate-polyacrylamide gels by the method of Laemmli [58]. Proteins were probed with rabbit anti-YAP1 monoclonal antibody (1:10000) (Cell Signaling Technologies), anti-DLCK1 (1:500) (Abcam), anti-BCL2 (1:500) (NeoMarkers), anti-BMI1 (1:10000) (Cell Signaling Technologies), anti-SOX2 (1:1000) (Cell Signaling Technologies), anti-c-MYC (1:500) (Santa Cruz Biotech Inc.), anti-TS (1:500) (Millipore), anti-α-tubulin (1:50000) (Santa Cruz Biotech Inc.), anti-GAPDH (1:100000) (Santa Cruz Biotech Inc.) and anti-β-actin (1:50000) (Sigma Aldrich). Horseradish peroxidase conjugated antibodies against mouse or rabbit (1:5000, Santa Cruz Biotech Inc.) were used as the secondary antibodies. Protein bands were visualized with autoradiography film using SuperSignal West Pico Chemiluminescent Substrate (Thermo Fischer). Western blot density was quantified using Image J software. All Western blots were repeated 3 times for quantification.

### Cell proliferation

Twenty-four hours after transfection, cells were replated in 96 well plates, at 1000 cells per well. Cell number was measured on days 1, 3 and 6 post transfection using WST-1 dye (Roche). Cells were incubated with 10 μl of WST-1 dye per 100 μl of media for 1 hour and absorbance was read at 450 and 630 nm. The O.D. was calculated by subtracting the absorbance at 630 nm from that at 450 nm.

### Cell cycle analysis

Twenty-four hours after transfection, cells were resuspended at 0.5 to 1×10^6^ cells/ml in modified Krishan buffer supplemented with 0.02 mg/ml RNase H (Thermo Fischer) and 0.05 mg/ml propidium iodide (Sigma-Aldrich). Stained cells were detected by flow cytometry and results were analyzed with Modfit LT software.

### Luciferase assay

A 441 nucleotide portion of the *YAP1* mRNA 3’-UTR containing a sequence complementary to the seed sequence of miR-15a was cloned into pMIR-Report Vector (Thermo Fischer) using forward primer: CGAGCTCTCTAAGGAGACACAT and reverse primer: CGACGCGTATATCAGATATAACA. A 973 nucleotide portion of the *DCLK1* 3’-UTR which containing two binding sites for miR-15a was cloned into pMIR-Report Vector using forward primer: CGAGCTCAGTCCTAGCTTAACC, and reverse primer: CGACGCGTCTGACACTGAGTAGA. Twenty-four hours before transfection, 1.5×10^4^ cells were plated in 96-well plate. 10 nM of miR-15a or control miRNA was transfected into these cells together with 100 ng of pMIR-Report-YAP1 or pMIR-Report-DCLK1 and 1 ng of Renilla luciferase plasmid pRL-SV40 (Promega) by DharmaFect Duo (Dharmacon, GELifeSciences) following the manufacturer's protocol. The luciferase assay was performed 24 hours after transfection by dual-luciferase reporter assay system (Promega). For each sample, firefly luciferase activity was normalized to Renilla luciferase activity and the inhibition by miR-15a was normalized to the control miRNA.

### 5-FU treatment and cytotoxicity assay

Twenty-four hours after transfection, HCT116 cells were plated in 96-well plates at 2000 cells per well in triplicate, in 100 ml of medium supplemented with 10% Dialyzed FBS (Thermo Fischer). After 24 hours, fresh media containing 5-FU alone (ranging from 2 to 7.5 mM), miR-15a precursor alone (ranging from 20 to 75 nM) or 5-FU and miR-15a together (at a constant ratio 1:100, with increasing concentrations of both compounds) was added, and cells were cultured for 72 hours. Cell viability was measured using the WST-1 assay, and concentration-dependent curves were generated based on the cell viability. The Combination Index (CI) as well as IC^50^ for each alone as well as in combination was calculated using CompuSyn software (www.combosyn.com).

### Lentiviral production

All the materials for lentivirus production were purchased from GeneCopoeia and production was carried out following the manufactures protocol. Briefly, 1.5×10^6^ 293T cells were plated in 10 cm dish with 10 ml of DMEM+10% FBS. Two days later, pEZX-MR03, a lentiviral plasmid, expressing hsa-miR-15a was transfected with Lenti-Pac HIV expression packaging kit. 48 hours later, virus was harvested and concentrated with Lenti-Pac lentivirus concentration solution. The titer of the virus was determined with Lenti-Pac HIV qRT-PCR titration kit. In addition, serial dilution of the virus (1 μl, 5 μl, 10 μl, 20 μl, 50 μl) was used to transduce 5×10^4^ spheroid growing HCT116 cells to determine the transduction efficiency. The lowest volume (20 μl) to achieve 100% positive expression was used to infect the cells for *in vivo* experiments.

### Sphere formation assay

HCT116 derived spheroid growing cells were dissociated into single cell suspension and plated at 1×10^5^ cells per well in non attaching 6 well plates. Cells were transfected with control or miR-15a. Six days after transfection, sphere formation was assessed and representative photos were taken (100x magnification). Sphere size was quantified using ImageJ software. Spheres were characterized as particles with an area over 500 pixels.

### Colony formation assay

HCT116 derived spheroid growing cells were dissociated into single cell suspension and plated at 1×10^5^ cells per well in non-attaching 6 well plates. 24 hours after transfection with control miRNA, miR-15a or Mimic-1, these cells were mixed in culture media with 30% Matrigel (Corning) and plated. Once gel was formed, additional media was added over the gel layer. 6 days after transfection, colony formation was assessed and representative photos were taken (100x magnification).

### Human cancer stem cell profiler

RNAs were extracted from spheroid growing HCT116 cells transfected with either miR-15a or control miRNA using TRIzol reagent (Thermo Fischer) in accordance with the manufacturer's protocol. RNAs were transcribed to first-strand cDNA using the RT^2^ First Strand Kit (Qiagen). Next, the cDNA was mixed with RT^2^ SYBR Green Mastermix (Qiagen), and this mixture was aliquoted into the wells of the Human Cancer Stem Cells RT^2^ Profiler PCR Array (Qiagen). Applied Biosystems 7500 Real-Time PCR machine was used for qRT-PCR (Applied Biosystems), and relative expression values were determined using the ΔΔCT method.

### Mouse Xenograft and colon cancer metastasis models

All animal procedures were approved by the Stony Brook University Institutional Animal Care and Use Committee (IACUC). Two days before injection, spheroid growing HCT116 cells were plated at 5×10^5^ cells/well in 6-well ultra low attachment plates. 20 μl of the virus or 100 pmol miRNA precursors was used to transduce or transfect cells. 48 hours later, cells were collected and resuspended at 1×10^6^ cells per ml in DMEM/F12 knockout media with 30% matrigel. Twelve week old NOD/SCID mice (Jackson Laboratories, Bar Harbor, MA, USA) were anesthetized by isoflurane inhalation. 100 μl of cell suspension was injected subcutaneously into both the left and right flank. The tumor size was measured using a caliper and tumor volume was calculated using the formula V = (length x width^2^)/2.

For the impact of Mimic-1 in colon cancer metastasis studies, mouse tumors were established via tail vein injection (2 × 10^6^) cells with HCT116 cells expressing luciferase reporter. Two weeks after injection, mice were treated via tail vein injection with 40 μg of control miRNA or Mimic-1 packaged using *in vivo*-jetPEI (Polyplus Transfection) or with 50 mg/kg of 5-FU (Sigma Aldrich). Mice were treated every other day for 2 weeks (8 times). Following treatment, mice were screened using IVIS Spectrum *In vivo* Imaging System (IVIS) (PerkinElmer). 5-FU treated mice were not imaged as they died from 5-FU toxicity (n=5).

### miRNA modifications

miR-15a mimics were modified by substituting uracil with 5-FU. Oligonucleotides with these modifications as well as the corresponding passenger strand were purchased from Dharmacon (GELifeSciences). We have used a control modified miRNA based on cel-miR-67 (UCACAACCUCCUAGAAAGAGUAGA) from *C. elegans* in which uracil has been replaced with 5-FU. The modified miRNA and the passenger strand were annealed prior to use for transfection.

### Statistical analysis

All experiments were repeated at least three times. All statistical analyses were performed with SigmaPlot software. The statistical significance between two groups was determined using Student's *t*-test. For comparison of more than two groups, one-way ANOVA followed by a Bonferroni-Dunn test was used. Data were expressed as mean ± standard deviation. (^*^ *p ≤* 0.05, ^**^ *p ≤* 0.01, ^***^ *p ≤* 0.001).

## SUPPLEMENTARY MATERIALS FIGURES AND TABLES



## Abbreviations

CSC: colon cancer stem cells
CLL: chronic lymphocytic leukemia
TCGA: The Cancer Genome Atlas
TS: thymidylate synthase
