# Supplementary material for: Modified miR-15a has therapeutic potential for improving treatment of advanced stage colorectal cancer through inhibition of BCL2, BMI1, YAP1 and DCLK1
Source: Oncotarget. 2017 Dec 19;9(2):2367–83. doi: 10.18632/oncotarget.23414 (PMC5788646; doi:10.18632/oncotarget.23414)
Supplement: Supplementary file 1 [file oncotarget-09-2367-s001.pdf]

Modified miR-15a has therapeutic potential for improving treatment of advanced stage colorectal cancer through inhibition of BCL2, BMI1, YAP1 and DCLK1

SUPPLEMENTARY MATERIALS

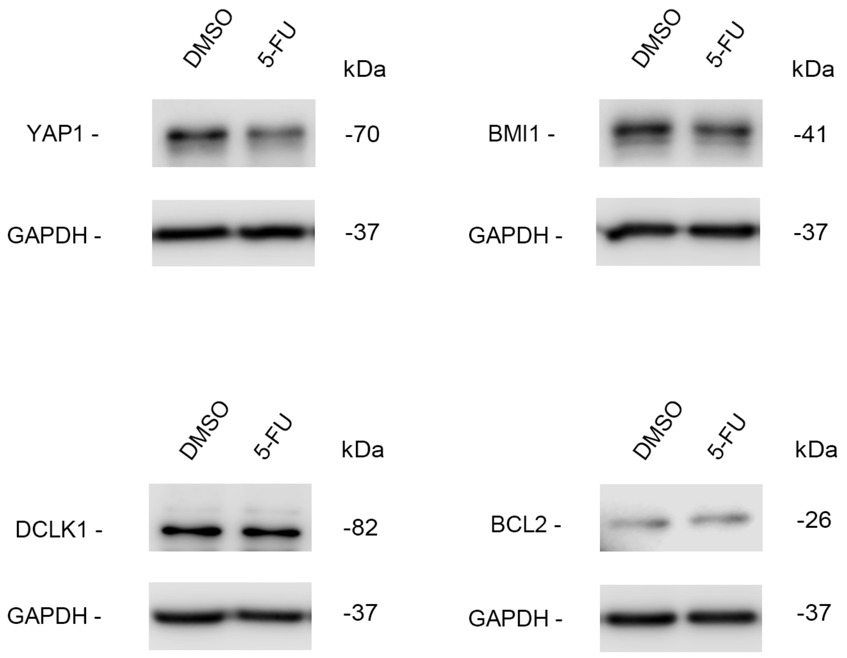

Supplemental Figure 1: 5-FU does not effect expression of miR-15a targets. Western blot indicates 5-FU alone (700 nM) has no significant effects on expression of the miR-15a target genes YAP1, BMI1, DCLK1 and BCL2.

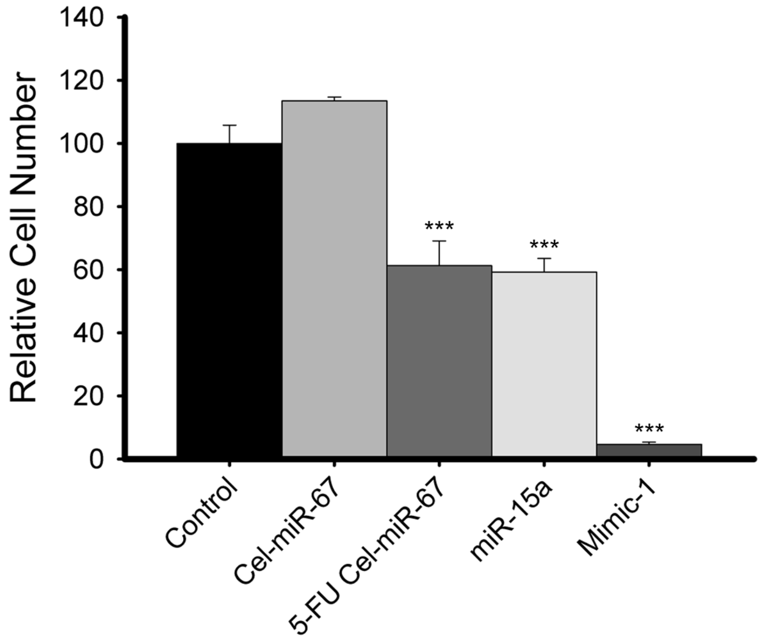

Supplemental Figure 2: The effects of Mimic-1 are not caused by 5-FU alone. Cell number 6 days following transfection is reduced 40% by a 5-FU modified control miRNA, *C. elegans* cel-miR-67, while Mimic-1 reduced cell number by 95%. (*** $p \leq 0.001$).

Supplemental Table 1: Human Cancer Stem Cells RT[2] Profiler PCR Array Expression Fold Change Following miR-15a Transfection

Symbol	Fold Change	Symbol	Fold Change	Symbol	Fold Change
ABCB5	0.28	GATA3	0.28	PECAM1	0.28
ABCG2	1.12	GSK3B	1.40	PLAT	0.28
ALCAM	1.30	HDAC1	1.20	PLAUR	0.78
ALDH1A1	0.28	ID1	1.38	POU5F1	2.76
ATM	0.91	IKBKB	1.81	PROM1	1.38
ATXN1	3.31	IL8	5.64	PTCH1	0.42
AXL	2.86	ITGA2	1.05	PTPRC	0.28
BMI1	1.61	ITGA4	0.28	SAV1	2.01
BMP7	1.27	ITGA6	1.32	SIRT1	1.58
CD24	2.06	ITGB1	1.34	SMO	1.14
CD34	0.28	JAG1	1.44	SNAI1	2.52
CD38	0.28	JAK2	1.08	SOX2	0.28
CD44	0.82	KIT	0.28	STAT3	0.89
CHEK1	0.71	KITLG	1.49	TAZ	1.24
DACH1	0.28	KLF17	0.49	TGFBR1	1.86
DDR1	1.17	KLF4	1.93	THY1	0.32
DKK1	0.99	LATS1	1.49	TWIST1	0.40
DLL1	0.69	LIN28A	0.49	TWIST2	0.28
DLL4	0.53	LIN28B	0.28	WEE1	0.70
DNMT1	1.19	MAML1	1.42	WNT1	0.28
EGF	1.61	MERTK	1.19	WWC1	2.65
ENG	0.29	MS4A1	0.28	YAP1	1.28
EPCAM	1.11	MUC1	1.05	ZEB1	0.70
ERBB2	1.63	MYC	2.02	ZEB2	0.28
ETFA	1.39	MYCN	0.28	ACTB	1.52
FGFR2	2.04	NANOG	1.67	B2M	1.26
FLOT2	1.25	NFKB1	1.60	GAPDH	0.68
FOXA2	0.28	NOS2	0.36	HPRT1	0.85
FOXP1	1.75	NOTCH1	2.15	RPLP0	0.92
FZD7	1.60	NOTCH2	0.91	HGDC	0.28
